# Insurance companies, climate and health justice

**DOI:** 10.2471/BLT.25.294116

**Published:** 2026-02-03

**Authors:** Hiroaki Matsuura, Takefumi Ueno

**Affiliations:** a Faculty of Tourism, Media, and Cultural Studies, Shoin University, 9-1 Morinosato-Wakamiya, Atsugi, Kanagawa 243-012, Japan.; b School of Management and Informatics, University of Shizuoka, Shizuoka, Japan.

Private sector commitments to global governance have grown substantially since the launch of the United Nations (UN) Global Compact.^1^ Within this evolving landscape, the commercial determinants of health framework^2^ offers a critical lens through which to examine how corporate strategies shape population health. Unlike other largely voluntary, UN-endorsed frameworks, the commercial determinants of health approach focuses on structural power imbalances, and calls for a dual strategy of enforceable regulations alongside voluntary corporate commitments to effectively protect public health.^3^

This approach is particularly relevant to the insurance sector, whose impact on climate and health outcomes remains under-recognized. In the health sector, insurance companies play an increasingly central role in health-care systems, not only in the United States of America, where the private sector dominates, but also across other countries of the Organisation of Economic Co-operation and Development. These companies shape provider networks, reimbursement models and health data use.^4^ Beyond the health sector, life, property and casualty insurance companies are similarly advancing public health by incentivizing safety, helping to prevent accidents and strengthening social and economic resilience. This positive outcome occurs because insurance companies shape incentives and standards that promote risk reduction and safer practices across society. Simultaneously, insurers serve as both enablers and investors in the fossil fuel industry.^5^ By underwriting legal, physical and reputational risks, insurers give otherwise unviable fossil fuel projects financial viability. Globally, they also manage over 40 trillion United States dollars (US$) in assets, with most tied to carbon-intensive industries.^6^ While insurers support disaster recovery and contribute to health system resilience, their current investment and underwriting practices raise concerns about long-term planetary and public health outcomes.

The Environmental, Social and Governance framework^7^ in global finance gained prominence with the 2006 launch of the UN Principles for Responsible Investment,^8^ which sought to integrate environmental, social and governance criteria across asset classes. Building on this foundation, insurance-sector initiatives such as the United Nations Environment Programme Finance Initiative Principles for Sustainable Insurance,^9^ together with cross-sector disclosure frameworks like the Task Force on Climate-related Financial Disclosures^10^ and the Taskforce on Nature-related Financial Disclosures,^11^ have shaped insurers’ approaches to sustainable governance, underwriting and investment practices.

Yet, despite these frameworks, the voluntary nature of commitments allows many large life, health and property and casualty insurers to continue supporting carbon-intensive industries through both underwriting and investment. Lloyd’s of London, for example, continues to insure fossil fuel projects at scale despite public commitments to the contrary, illustrating how environmental, social and governance language often masks inaction or selective disclosure.^5^ In the United States, major insurers such as State Farm and Berkshire Hathaway, both of which are also involved in health insurance, continue to hold substantial investments in fossil fuel companies.^5^ This contradiction highlights a broader ethical dilemma: insurers in multiple sectors profit from or perpetuate the very risks they insure against. Property and casualty insurers enable climate-related disasters through fossil fuel underwriting, while life and health insurers invest in industries that worsen the health outcomes they are meant to protect against.

The commercial determinants of health framework helps situate insurance companies as structural actors influencing health, emphasizing the need for regulatory action over voluntary compliance by reframing climate change as a pressing, human-centred crisis, driven in part by corporate practices. For example, tobacco control succeeded because it replaced voluntary, partnership-based environmental, social and governance approaches with binding legal accountability and the exclusion of industry influence.^12^ The WHO Framework Convention on Tobacco Control (FCTC) established a binding international treaty that obliges governments to regulate and protect public health from corporate interference, particularly through Article 5.3, which requires parties to protect public health policy from the industry’s commercial and vested interests, with guidelines limiting and requiring transparency of any interactions.^13^ This legal architecture, supported by civil society mobilization and global coordination, exposed and delegitimized the industry’s manipulation of science and policy, although implementation has been uneven and contested in many settings.^12^ As a result, governments reasserted their duty to protect health over corporate interests, marking a normative and structural shift from voluntary ethics to enforceable governance that other sectors such as fossil fuels or ultra-processed foods have yet to replicate.

By contrast, the fossil-fuel industry is deeply embedded in global economies, producing diffuse, system-wide health harms that complicate both regulation and divestment. In some contexts such as Texas, United States, laws like Senate Bill 13 restrict State contracts and investments with financial firms expected to boycott energy companies, indirectly discouraging insurers and asset managers from divesting from fossil fuels and exemplifying regulatory capture. The commercial determinants of health framework advances beyond the environmental, social and governance framework by reframing commercial influence as a determinant of health that requires binding governance, transparency and power analysis rather than voluntary self-regulation. The framework also clarifies why tobacco-free finance remains constrained in countries where governments maintain ownership of or fiscal dependence on tobacco revenues, creating structural conflicts of interest that undermine both the implementation of FCTC Article 5.3 and broader public health protection. Together, these dynamics illustrate how governance failures and structural conflicts of interest constrain financial actors, including insurers, from disengaging from harmful industries despite well-documented health risks.

Within the insurance sector, these constraints manifest as a growing internal contradiction. Many leading insurers still finance activities that exacerbate climate-related health risks, even as extreme weather drives record claims, rising premiums and shrinking coverage zones that threaten the insurance model itself. The Geneva Association links heat and wildfires to major insured losses and health harms, showing how mounting payouts and reduced protection erode public trust.^14^ Continued fossil fuel investments thus pose mounting financial and ethical risks, raising questions about whether fiduciary duty (that is, the obligation to act in beneficiaries’ best interests) prioritizes long-term societal resilience or short-term returns. Several major insurers or reinsurers such as AXA, Allianz, Swiss Re and Munich Re have adopted or expanded fossil-fuel exit policies in recent years, notably by phasing out coal underwriting and restricting new oil and gas projects. However, these policies remain partial and uneven across companies.^5^ Where withdrawal occurs rapidly or without coordination, it may reduce investment returns, constrain underwriting capacity and contribute to higher insurance premiums. Such dynamics can create transitional inequities as early movers absorb financial costs while slower actors retain competitive advantage. Rising premiums are likely to disproportionately affect low-income households, while the withdrawal of coverage may contribute to the emergence of insurance deserts in high-risk areas. In addition, uncoordinated divestment may pose risks to the financial stability of national insurance schemes that remain reliant on fossil-linked assets.

These financial and distributional consequences bring fiduciary duty to the forefront as a central governance question for the insurance sector. Fiduciary duty is increasingly recognized as both a legal and ethical foundation requiring insurers to address climate risk through their investment and underwriting practices. As scientific evidence of climate-related threats grows, an increasing number of scholars argue that insurers who fail to incorporate these risks, particularly by enabling carbon lock-in, whereby continued support for carbon-intensive systems delays decarbonization, may be falling short of evolving fiduciary standards.^13^ Once narrowly defined as the pursuit of short-term financial returns, fiduciary responsibility has expanded to encompass long-term systemic risks, including threats to the health and well-being of both current and future populations. This broader interpretation calls for the integration of principles of intergenerational and international justice, particularly in addressing health inequities associated with climate-driven premium increases and continued exposure to high-emission sectors. To support a fair transition, insurers must not only redirect capital away from harmful industries but also expand equitable, climate-resilient coverage through inclusive risk-sharing mechanisms.

This reframing broadens the concept of material risk to include global health impacts, making inaction financially questionable and ethically problematic. A series of cases, *Spence* v. *American Airlines* (2023), *ClientEarth* v. *Shell Board of Directors* (2023), *McRitchie* v. *Zuckerberg* (2023) and *Milieudefensie et al.* v. *Shell* (2019), collectively illustrate the expanding scope of fiduciary duty, emphasizing the obligation of companies to address long-term climate and environmental risks to protect shareholder value and future financial stability.^15^ Courts may increasingly interpret fiduciary duty to require the integration of climate risk, even in the absence of new regulatory mandates. In this way, fiduciary duty may offer a third way, neither reliant solely on government regulation nor confined to voluntary environmental, social and governance initiatives. Instead, fiduciary duty embeds climate accountability within existing legal obligations, encouraging insurers to act in the long-term interests of their beneficiaries, society and the planet.

Insurers can no longer be regarded as neutral financial intermediaries. They are integral to commercial determinants of climate and health outcomes, with the potential to either slow or advance a just transition. Their responsibilities extend beyond portfolio management and shareholder returns to include safeguarding the environmental and social foundations of public health. While voluntary environmental, social and governance initiatives have merit, binding measures are now essential. Governments should embed fiduciary and climate accountability in financial regulation, integrating health risk into solvency and disclosure standards. Insurers must align portfolios with net-zero targets, disclose carbon exposure and extend coverage to climate-vulnerable groups. Shareholders and communities alike should use stewardship and advocacy to demand fair, transparent and climate-consistent governance. Collectively, these actions would recast insurers as guarantors of intergenerational and planetary health.
